# Enhancing Alfalfa (*Medicago sativa*) Seed Yield: The Effect of Honey Bee (*Apis mellifera*) Supplementation and Efficiency of Other Pollinators

**DOI:** 10.3390/biology14060599

**Published:** 2025-05-25

**Authors:** Kamran Ejaz, Mudssar Ali, Fawad Zafar Ahmad Khan, Raimondas Mozūratis

**Affiliations:** 1 Institute of Plant Protection, Muhammad Nawaz Shareef University of Agriculture Multan, Multan 60000, Pakistan; kamran3194@gmail.com (K.E.); fawad.zafar@mnsuam.edu.pk (F.Z.A.K.); 2 Department of Outreach and Continuing Education, Muhammad Nawaz Shareef University of Agriculture Multan, Multan 60000, Pakistan; 3 Department of Zoology, Stockholm University, SE-10691 Stockholm, Sweden; 4 Laboratory of Chemical and Behavioural Ecology, The State Scientific Research Institute Nature Research Centre, LT-08412 Vilnius, Lithuania

**Keywords:** bees, fodder, foraging behaviour, tripping behaviour

## Abstract

Alfalfa (*Medicago sativa*) is an important fodder crop with 16–25% protein and 20–30% fibre content. It requires pollinators for seed development through a process known as “tripping”. Honey bees are preferred for their easy management and mass rearing to enhance pollination. In a field experiment, we tested three treatments: two honey bee colonies (moderately supplemented), three honey bee colonies (highly supplemented), and a control with no honey bee colonies. The colonies European honey bee, *A. mellifera*, were used, starting at the flowering stage. We assessed the abundance, foraging, and tripping behaviour of pollinators, as well as the number of seeds per pod, pod weight, and seed weight. Honey bees were the most abundant in supplemented plots, followed by solitary bees, with increased honey bee visitation in the second year. *Megachile cephalotes*, *M. hera*, and *A. mellifera* were the most efficient pollinators regarding seeds per pod and seed weight. The plot with two honey bee colonies had a higher seed yield than the one with three colonies. Conservation of these pollinators and the introduction of managed *A. mellifera* colonies can lead to sustainable alfalfa seed production. Our findings suggest that honey bee supplementation, along with conservation efforts for solitary bees, can enhance pollination and seed yield in alfalfa.

## Simple Summary

Alfalfa is a widely grown crop used as an animal feed because of its protein and fiber content. However, alfalfa plants cannot produce seeds without the assistance of flower-visiting insects, which transfer pollen between flowers. In this study, we tested whether placing honey bee colonies near alfalfa fields could improve pollination and increase seed production. We compared three types of field setups: one without honey bees, one with two colonies, and one with three colonies. We recorded how often insects visited the flowers, how they behaved while feeding, and how many seeds were produced. Honey bees were the most common visitors in the fields where hives were placed, although native wild bees also contributed significantly to pollination. We found that the setup with two honey bee colonies produced more seeds than the setup with three. This suggests that more bees do not always lead to better results. Our results also showed that both honey bees and native wild bees played a role in seed development. This research shows that a balanced use of managed honey bees has the potential to improve seed production in alfalfa.

## 1. Introduction

With the increasing human population, the demand for dairy and meat products is increasing; therefore, the livestock industry is expanding. The healthy development of livestock relies on the consistent availability of high-quality fodder. Pakistan produces approximately 55.47 million tons of fodder annually [1]. The main fodder crops in Pakistan include sorghum (*Sorghum bicolor* L.) (Poales: Poaceae), pearl millet (*Pennisetum glaucum* L.) (Poales: Poaceae), maize (*Zea mays* L.) (Poales: Poaceae), cowpeas (*Vigna unguiculata* L.) (Fabales: Fabaceae), guar (*Cyamopsis tetragonoloba* L.) (Fabales: Fabaceae), berseem clover (*Trifolium alexandrinum* L.) (Fabales: Fabaceae), alfalfa (*Medicago sativa* L.) (Fabales: Fabaceae), oats (*Avena sativa* L.) (Poales: Poaceae), and ryegrass (*Lolium perenne* L.) (Poales: Poaceae).

*M. sativa* is an important fodder crop, containing 20–30% fibre and 16–25% protein [2]. Due to its high nutritional value and yield, alfalfa is known as “the queen of fodders” [3]. It is a perennial forage legume, a deep-rooted crop with multiple cuttings and a fast-growing nature, characterised by purple flowers [4,5]. The inflorescence of alfalfa is trifoliolate, 1.0–2.5 cm long, and 0.3–1.6 cm wide. Papilionaceous flowers remain closed during anthesis and only open after being “tripped” by a pollinator, making alfalfa highly dependent on entomophilous pollination. Furthermore, alfalfa has a reproductive system that is primarily self-incompatible and self-sterile [6]. The fruit of alfalfa is a polyspermous pod. Small, sickle-shaped, or corkscrew-coiled alfalfa pods typically have one or more coils, with a maximum of five. Alfalfa seeds are tiny, reniform, angular, or oval-shaped, with colours ranging from yellow to light brown and greyish brown [7].

In Pakistan, alfalfa is cultivated as a mixed crop for fodder and seed production. After multiple cuttings, the crop is left to mature for seed production [8]. Pakistan has a low output of alfalfa fodder and seeds. The total cultivated area under fodder crops is projected to exceed two million hectares, with an annual fodder production of 55.4 million tons. However, to meet the feeding requirement, this amount of fodder is insufficient [9]. The low production levels can be attributed to the unavailability of high-quality seeds and the farmers’ limited awareness of advanced crop production technologies [10].

Effective pollination in alfalfa requires a tripping mechanism, where pollinators must physically force and open flower parts to release pollen grains, enabling successful seed production [11]. There are different pollinators of the alfalfa crop, among which bees are considered the primary pollinators [12,13,14,15]. Managed honey bee *Apis mellifera* L. (Hymenoptera: Apidae) ranks as the most frequently visiting single species in many crops, including alfalfa [16]. *A. mellifera* is an important pollinator of alfalfa crops mainly due to its ability to forage for more extended periods up to 11 h per day. This prolonged foraging activity improves its effectiveness in pollinating alfalfa, which makes it particularly valuable for optimising seed yield [17].

Supplementary bee pollination has been used to enhance the productivity and quality of various crops. For example, in melons, supplemental pollination by managed solitary bees and honey bees enhanced the fruit set, fruit size and yield parameters of the crop [18]. Moreover, another study on oil tree peonies has shown that supplementary honey bee pollination can significantly enhance fruit growth rate and yield [19]. In the same study, managed honey bees in controlled pollination settings have increased seed production and oil content [19]. Often, a single pollinator species is insufficient for efficient pollination, therefore, the presence of other pollinators is necessary [20,21,22].

To increase alfalfa crop yield, farmers use bee colonies and place them near crops for the best pollination [23]. For optimal alfalfa seed production, 4–6 colonies have been recommended per hectare [24]. Although honey bees are highly effective in increasing pollination rates and seed yields due to their ability to be mass-reared and easily managed, they are less efficient at pollinating alfalfa flowers compared to some solitary bees [25,26]. However, the management of solitary bees requires year-round maintenance of floral resources as well as nesting sites [27]. For example, *Megachile* spp. are highly efficient in tripping alfalfa flowers, yet they present unique management challenges due to their nesting needs and lower tolerance to environmental disturbances [27]. Moreover, due to climate change, the number of native solitary bees is expected to decrease [28,29]. In these scenarios, honey bee supplementation could serve as a quick option to improve pollination services in alfalfa crops.

The increased presence of *A. mellifera* can lead to enhanced competition for floral alfalfa resources, potentially affecting foraging behaviour and the population dynamics of local pollinators [30]. Despite this, when combined with conservation efforts for solitary bees, honey bee supplementation can create a balanced approach that enhances overall pollination efficiency and seed production in alfalfa fields [30].

Little is known about the effect of *A. mellifera* supplementation on foraging behaviour and the abundance of local pollinators in alfalfa fields. To our knowledge, only one study has compared the single-visit efficiency of native solitary bees in alfalfa crops in Pakistan [31]. No previous study has reported the role of *A. mellifera* supplementation in increasing the seed yield of alfalfa crops. Therefore, the current study was designed to investigate the optimal number of honey bee colonies for alfalfa seed production, considering the presence of local pollinators. We hypothesise that supplemental honey bee pollination significantly enhances alfalfa seed yield in response to pollination deficits. Based on this hypothesis, we predicted that alfalfa plots with supplemental honey bee pollination would yield higher seed yields than those without supplementation.

## 2. Materials and Methods

### 2.1. Study Site

The research was conducted at the experimental farm of Muhammad Nawaz Shareef University of Agriculture, Multan, Punjab, Pakistan (30.1575° N, 71.5249° E) over two seasons, including the years 2021–2022 and 2022–2023. The experimental crop was Alfalfa (*Medicago sativa* L.). Alfalfa cultivar ‘*Sargodha 2000*’ was sown at a rate of 8 kg per acre over a three-acre area on 8 November 2021 (first year) and 10 November 2022 (second year). The climate in Multan is subtropical, characterised by dry and hot summers, mild winters, and an average annual rainfall of 175 mm [32]. The minimum and maximum temperatures in this area in summer are 24 °C and 42 °C, respectively, while in winter, the range is between 4.5 °C and 22 °C [33,34]. The experimental plots and alfalfa sowing were managed homogeneously to exclude any variance that might impair the experiment’s analytical comparisons. Standard pest management practices (use of neem-based biopesticides) and agronomic practices (thinning and two cuttings) were implemented for the alfalfa crop, and manual weed removal was also performed.

### 2.2. Experimental Design and Layout

**Figure 1:**
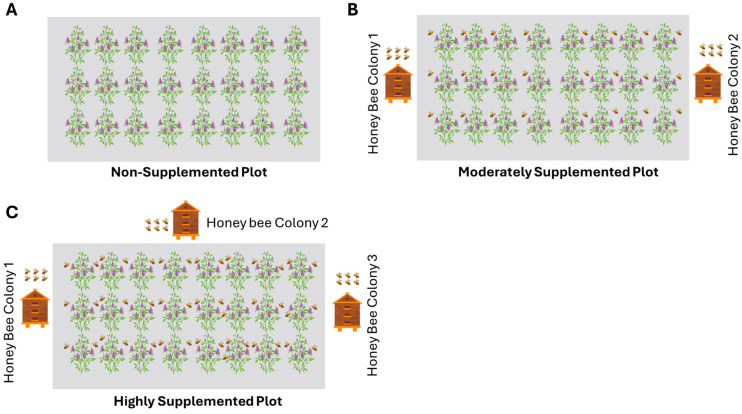
Experimental layout showing three pollination treatments in alfalfa fields: (**A**) Non-supplemented plot with no honey bee colonies, (**B**) Moderately supplemented plot with two *Apis mellifera colonies* placed at the field edges, and (**C**) Highly supplemented plot with a three *A. mellifera* colonies surrounding the field. The illustration represents the relative abundance of honey bees and colony placement across the treatments to assess the impact of honey bee supplementation on pollinator activity and seed set.

Three plots (each measuring 4046.86 m^2^) were established, which were at least 1 km apart from each other, and named as a highly supplemented block, a moderately supplemented block, and a non-supplemented plot. We used the distance measurement tool available in Google Maps to ensure that all selected experimental fields were at least 1 km apart. Three honey bee colonies were introduced into a highly supplemented block, while two colonies were placed in a moderately supplemented block (Figure 1). No colonies were introduced into the non-supplemented block, which was the control in both years. All honey bee colonies were healthy, with no signs of disease or pest infestation observed during or after the experiment. Throughout the experiment, all colonies were maintained under standard apicultural practices. They were provided with sugar syrup (1:1 ratio of sugar and water) as a supplemental food source. We also conducted routine inspections to monitor colony health, queen presence, and overall brood development. We did observe any absconding or swarming behavior during or after the experiment. Standard agronomic practices were followed for growing the alfalfa crop [35]. *A. mellifera* colonies, each housed in hives containing 10 frames, were used for this experiment. Each hive measured 20 cm (length) × 30 cm (height) × 16 cm (width), and the hive cover measured 20 cm × 16 cm × 4 cm. Each colony had an estimated strength of ~25,000–30,000 worker bees at the start of the experiment. The colonies were purchased from a progressive beekeeper in Layyah, Punjab, Pakistan, and were transported at night on a mobile vehicle due to the low activity of the bees. The bee colonies were then placed on an iron stand under the shade of a tree in the experimental farm of Muhammad Nawaz Shareef University of Agriculture, Multan, Punjab, Pakistan. For moderate honey bee supplementation, two hives were placed diagonally opposite each other, facing southeast. For high honey bee supplementation, three hives were arranged in a triangular formation, with two colonies aligned on one side and the third colony placed in the middle on the opposite side, facing southeast.

### 2.3. Abundance of Honey Bees and Other Insect Pollinators

The abundance of honey bees and other insect pollinators was measured using a one-square-meter iron frame placed in ten randomly selected quadrats per plot during the flowering period (April–May). The one-square-meter iron frame was randomly tossed to determine the observation area by dividing the experimental plot into units, assigning serial numbers to the units and using a random number generator to select places for the iron frame. After the frame landed, a one-minute pause was observed to ensure that pollinator activity had returned to normal. After the data collection from that area, the frame was tossed again while standing at the previous data point, and this process was repeated. The frame was placed directly on the ground to prevent any movement that could hinder the observation process. Insect pollinator counts were conducted twice weekly at four-hour intervals (06:00, 10:00, 14:00, and 18:00) throughout the flowering season (April–May) in both study years. Abundance was assessed by observing each quadrat for five minutes and recording the number of insect visitors [36].

### 2.4. Foraging Behavior

The duration of stay for honey bees and other insect pollinators was measured by visual observation of how long each pollinator stayed on a single raceme during a 60-s duration. The duration was recorded by using a digital stopwatch (XL-011, Binloo, China). The observer stood at least 0.3 m away from the alfalfa plant. During the observation period, care was taken to ensure that the observer remained still and avoided any unnecessary movement to prevent disturbance to pollinator activity. The visitation rate was calculated by counting the number of racemes visited by honey bees and other insect pollinators within 60 s. Visual observations were conducted twice weekly throughout the flowering season (April–May) at four different times of day: 06:00, 10:00, 14:00, and 18:00 h, to capture variation in pollinator activity [25,37].

### 2.5. Tripping Behaviour of Honey Bees and Other Pollinators

Tripping behaviour of insect pollinators was assessed through counting: (1) the number of flowers tripped per raceme (exposure of anthers upon pollinator interaction), and (2) the number of already tripped flowers visited per raceme (already exposed anthers due to a previous pollinator interaction) [38,39]. A tripped alfalfa flower was identified by the visible stigmas and anthers. In contrast, untripped flowers remained closed, with the reproductive structures not yet exposed. Data were collected twice a week throughout the entire flowering season of alfalfa.

### 2.6. Single-Visit Seed Set Efficiency and Reproductive Success of Alfalfa

To measure the single-visit seed set efficiency, the observer placed himself at least 0.3 m away from an unopened flower (un-tripped) and waited for a pollinator to trip it. After being tripped by a specific insect, the flower was caged using a butter paper bag (3 cm × 3 cm) and tagged with the name of the pollinator; the remaining flowers on that raceme were removed. Ten pollinators of the same species were each allowed a single visit to different flowers. After the seed ripened, the cages were removed, and the pods were harvested at maturity in June. After harvesting, the pod weight was measured using digital weighing balance. After weighing, pod was crushed manually to harvest the seeds. Following hand crushing, the seed weight was measured using digital weighing balance. Following the weight measurements, the number of seeds was counted in each floret. Single visits of seven pollinator species were assessed in the first year of the trial, as the pods visited by the two pollinator species, *Amegilla* sp. and *E. aeneus*, were damaged due to rainfall, and no seed was recovered. Moreover, no data regarding single-visit efficiency were collected during the second year.

### 2.7. Seed Yield

To assess the effect of honey bee supplementation on seed yield, seed weight per square meter was measured across three treatments: highly supplemented, moderately supplemented, and non-supplemented plots. We selected ten quadrats of 1 m^2^ with similar plant densities and marked them across the entire acre. These same plants were then used to assess reproductive success and record seed weight (in grams). Seed weight was then compared across treatments to evaluate the impact of honey bee supplementation on overall seed yield.

### 2.8. Statistical Analysis

To estimate abundance, the number of different insect pollinators visiting the flowers was analysed in 2022 and 2023. For stay time, the average time spent by each pollinator in seconds was compared between the two years. For the visitation rate, the mean number of flowers visited per minute was analysed for 2022 and 2023. For tripping efficiency, the average number of flowers tripped versus already-tripped flowers was recorded for each bee species and analysed separately for the two years. The mean seed weight per m^2^ across three honey bee supplementation treatments was also analysed separately each year. The single-visit efficiency of individual bee species was measured as the mean pod weight, mean seed weight, and mean seed number per visit of each bee species, and these metrics were analysed annually. We used the Shapiro–Wilk test to check whether the data followed a normal distribution and applied a log transformation to achieve normality. Analysis of variance (ANOVA) was performed, and means were compared using the Tukey test (Statistix 8.1).

## 3. Results

### 3.1. Abundance of Insect Pollinators

**Figure 2:**
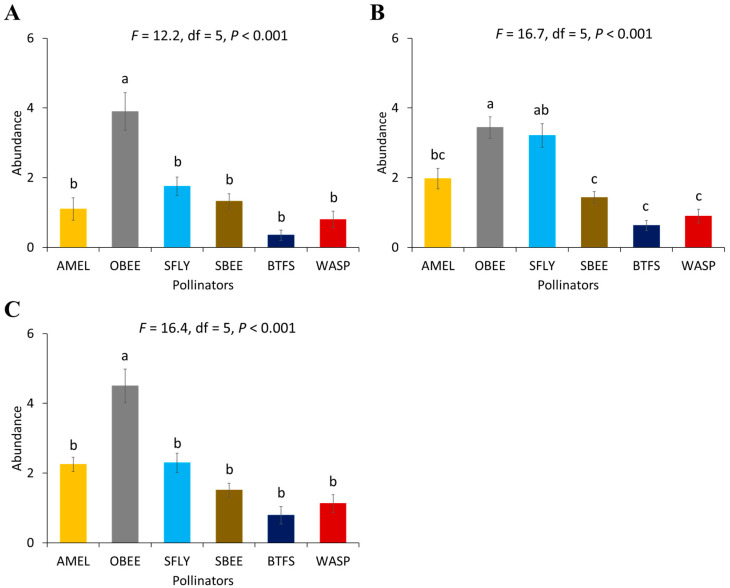
Abundance of different pollinator species visiting lucerne flowers in 2022: (**A**) non-supplemented block, (**B**) moderately supplemented block, and (**C**) highly supplemented block. Standard error bars with the same letters are not significantly different (Tukey–Kramer test, α  =  0.05). Pollinator group abbreviations: AMEL—*Apis mellifera*, OBEE—other honey bees; SFLY—syrphid flies; SBEE—solitary bees; BTFS—butterflies; WASP—wasps.

There was a significant difference in the abundance of pollinator species in all the non-supplemented block (*F* = 12.2, df = 5, *p* < 0.001), moderately supplemented block (*F* = 16.7, df = 5, *p* < 0.001), and highly supplemented block (*F* = 16.4, df = 5, *p* < 0.001) during the first year (Figure 1). Similar differences were found in the second year in non-supplemented block (*F* = 20.3, df = 5, *p* < 0.001), moderately supplemented block (*F* = 20.6, df = 5, *p* < 0.001), and highly supplemented block (*F* = 46.1, df = 5, *p* < 0.001) (Figure 2). In the first year, the abundance of other honey bees (*A. dorsata* and *A. florea*) was significantly higher in the highly supplemented block where three honey bee colonies were kept for supplementary pollination and in the non-supplemented block compared to the abundance of other pollinator species (Figure 1A,C). Syrphid flies (*E. aeneus* and *E. arvorum*) were also significantly higher than solitary bees (*M. cephalotes*, *M. hera*, and *Eucera* sp.), butterflies and wasps in the moderately supplemented block where two honey bee colonies were installed for supplementary honey bee pollination. The abundance of European honey bees (*A. mellifera*) was significantly greater in the moderately supplemented block than in other supplementary treatments (Figure 2).

**Figure 3:**
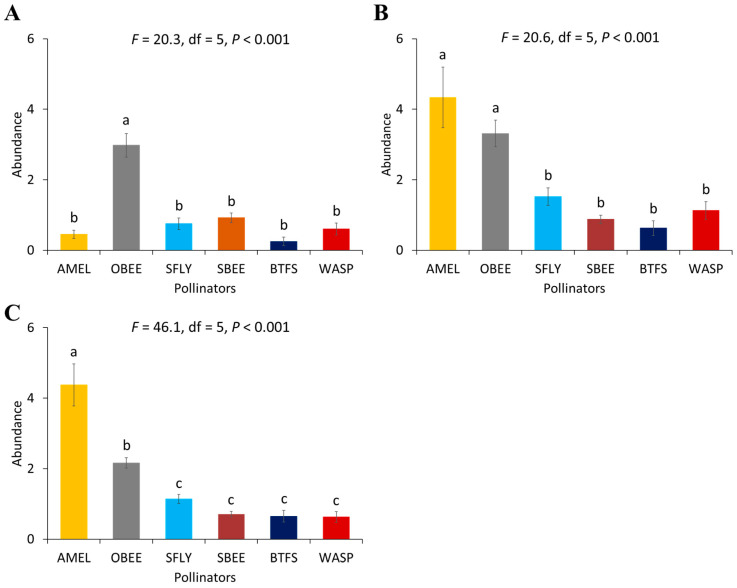
Abundance of different pollinator species visiting lucerne flowers in 2023: (**A**) non-supplemented block, (**B**) moderately supplemented block, and (**C**) highly supplemented block. Standard error bars with the same letters are not significantly different (Tukey–Kramer test, α  =  0.05). Pollinator group abbreviations: AMEL—*Apis mellifera*, OBEE—other honey bees; SFLY—syrphid flies; SBEE—solitary bees; BTFS—butterflies; WASP—wasps.

In the second year, the abundance of pollinator species was lowest where no honey bee hives were kept for supplementary pollination. The highest abundance of European honey bees (*A. mellifera*) was recorded in the moderately supplemented block compared to other blocks with varying levels of supplementation. The abundance of syrphid flies, solitary bees, butterflies, and wasps was not significantly different from one another in all of the supplementary pollinated plots in the second year (Figure 3).

### 3.2. Stay Time

**Figure 4:**
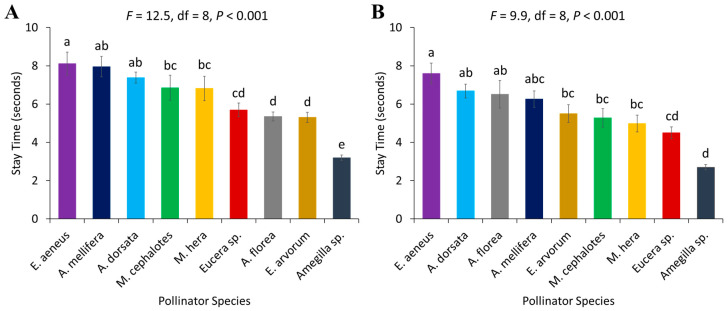
Stay time (time spent per flower) of different pollinator species in two years, (**A**) 2022 and (**B**) 2023. Standard error bars with the same letters are not significantly different (Tukey–Kramer test, α  =  0.05).

The time spent per flower by individual pollinator species differed significantly between the years 2022 (*F* = 12.5, df = 8, *p* < 0.001) and 2023 (*F* = 9.9, df = 8, *p* < 0.001). The stay time of *E. aeneus* was significantly higher than that of all other pollinator species in both years, while *Amegilla* sp. were significantly less frequent visitors in both years. There was no significant difference in the stay time of *A. mellifera*, *A. dorsata*, *M. cephalotes*, *M. hera* and *Eucera* sp. in both years. *A. florea* and *E. arvorum* were significantly lower than all other insect pollinators except *Amegilla* sp. (Figure 4).

### 3.3. Visitation Rate

**Figure 5:**
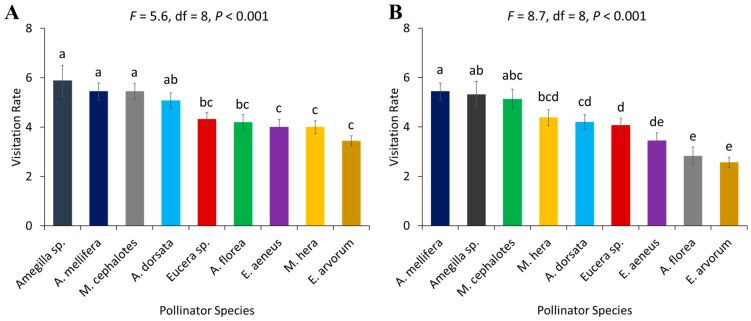
Visitation rate (number of flowers visited per minute) of different pollinator species in two years, (**A**) 2022 and (**B**) 2023. Standard error bars with the same letters are not significantly different (Tukey–Kramer test, α  =  0.05).

The number of visited flowers per minute by each pollinator species was significantly different in 2022 (*F* = 5.6, df = 8, *p* < 0.001) and 2023 (*F* = 8.7, df = 8, *p* < 0.001). The visitation rates of *Amegilla* sp., *A. mellifera*, and *M. cephalotes* were significantly higher than all other recorded pollinator species in both years. *Eucera* sp., *A. florea* and *E. aeneus* were not significantly different from each other and had a lower visitation rate as compared to other pollinator species. *E. arvorum* had the significantly lowest visitation rate of all pollinator species in both years (Figure 5).

### 3.4. Tripping

**Figure 6:**
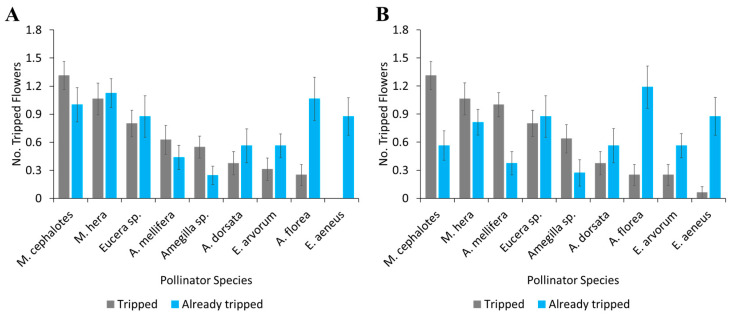
Tripping trends in alfalfa flowers (tripped vs. already-tripped flowers) by different pollinator species in (**A**) 2022 and (**B**) 2023. Error bars indicate standard error.

The tripping behaviour of insect pollinators differed significantly between the two years: 2022 (*F* = 11.19, df = 8, *p* < 0.001) and 2023 (*F* = 11.83, df = 8, *p* = 0.004). *Megachile* sp. was the most effective pollinator in terms of tripping efficiency, with tripping by *M. cephalotes* and *M. hera* being significantly greater than that of all other pollinator species. The number of tripped flowers due to the visits of *A. mellifera* was higher than that of other solitary and honey bees, followed by *Megachille* sp. bees. The number of already visited tripped flowers was higher in *A. florea,* followed by solitary bees (*M. hera*, *M. cephalotes*, *Eucera* sp.) in both years of data recordings (Figure 6).

### 3.5. Seed Weight

**Figure 7:**
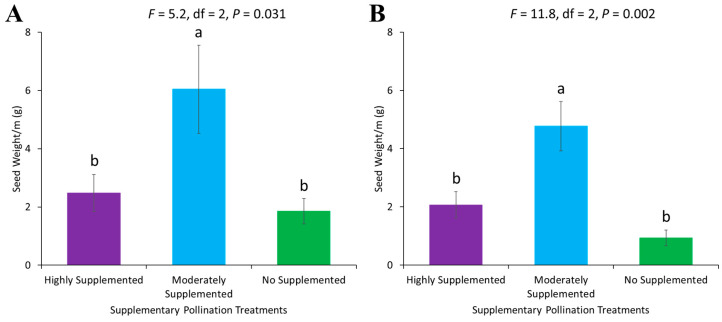
Seed weight (grams) per square meter in different supplementary pollination treatments in (**A**) 2022 and (**B**) 2023. Standard error bars with the same letters are not significantly different (Tukey–Kramer test, α  =  0.05).

Seed weight per square meter differed significantly across various supplementary treatments in 2022 (F = 5.2, df = 2, *p* = 0.031) and 2023 (F = 11.8, df = 2, *p* = 0.002). Seed weight was highest in the moderately supplemented block, where two honey bee colonies were placed for supplementary pollination. There was no significant difference in seed weight per square meter in the highly supplemented and non-supplemented blocks (Figure 7).

### 3.6. Single-Visit Seed Set Efficiency

**Figure 8:**
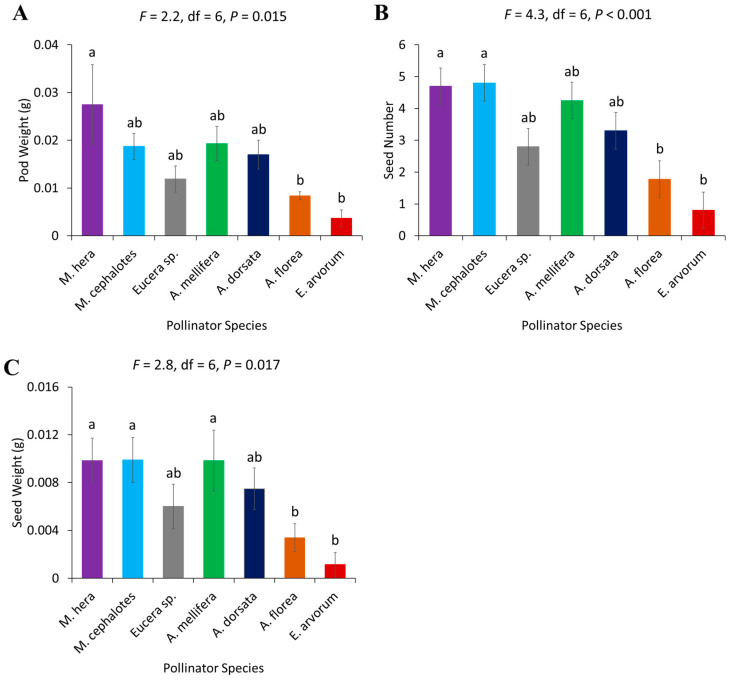
Yield parameters after a single visit by different pollinator species in a lucerne crop: (**A**) pod weight in grams, (**B**) seed weight in grams, and (**C**) seed number per pod. Standard error bars with the same letters are not significantly different (Tukey–Kramer test, α  =  0.05).

There was a significant difference among the pollinator species in terms of pod weight (*F* = 2.2, df = 7, *p* = 0.015), seed weight (*F* = 2.8, df = 6, *p* = 0.017), and number of seeds produced (*F* = 4.3, df = 6, *p* < 0.001) of the tested pollinator species in a single visit. Single-visit efficacy showed that *M. hera* and *M. cephalotes* were the most effective insect pollinators, followed by *A. mellifera*. Syrphid flies (*E. arvorum*) were the least effective in terms of pod weight, seed weight and seed number (Figure 8). Moreover, the Pearson’s correlation analysis showed that flower tripping by pollinators was positively and significantly associated with all measured seed yield parameters (Table 1). Statistically significant correlation was observed between flower tripping and pod weight (*r* = 0.2575, *p* = 0.0433). Similarly, flower tripping showed a stronger correlation with the number of seeds per pod (*r* = 0.3605, *p* = 0.0040) and seed weight (*r* = 0.3347, *p* = 0.0078). Among the seed parameters themselves, significant positive correlations were found. Pod weight was significantly correlated with both the number of seeds (*r* = 0.4812, *p* < 0.001) and seed weight (*r* = 0.5286, *p* < 0.001). The strongest correlation observed was between the number of seeds and seed weight (*r* = 0.8907, *p* < 0.001) (Table 1).

## 4. Discussion

The present research showed that *Megachile cephalotes* and *M. hera* were the most prevalent visitors of alfalfa flowers, followed by syrphid flies, solitary bees, honey bees, and wasps, while lepidopterans were sporadically observed in non-supplemented fields. Some previous studies have reported variable results regarding the effects of supplementary honey bee pollination on the yield of different crops. In this study, we observed that the bee population affects pollination efficacy on the crop, and ultimately, yield differences were observed in different supplemented fields. Honey bee colonies were placed around the alfalfa field to test the varying pollination efficacy by *A. mellifera* according to the supplemented treatments, while in one plot, no honey bee hive was installed. It was found that the abundance was higher in honey bee-supplemented plots as compared to non-supplemented plots. Within the supplemented plots, the abundance of *A. mellifera* was higher with the moderately supplemented treatment compared to the higher supplementary treatment. Zhang et al. [19] reported that bees at lower abundances pollinated more efficiently than those at higher abundances. While it is generally assumed that a higher number of supplemented honey bees will enhance crop pollination and production, some studies have shown that this is not always the case [17,40]. Sufficient bee pollination could ensure stable yield and maintenance of genetic variability of crop species [41]. It is also reported that the highest yield was obtained when the optimum number of *A. mellifera* (6–8 bees per 1000 flowers) was present for pollination of kiwifruit (*Actinidia chinensis*) [42]. They also revealed that when there were 60 flowers in a square meter, pollination services by *A. mellifera* were better with 22 honey bees present per square meter, compared to 44 honey bees per square meter [42].

In this study, we found that the highly supplemented alfalfa field yielded less than the moderately supplemented field. This suggests that foraging resources become limited when a high number of supplemented honey bees are introduced to enhance pollination. Similar results have been reported in a study where increased pollen deposition on the stigma of a flower, due to frequent visits by *A. mellifera*, did not affect the drupelet set of raspberries. However, it is suggested that a higher visitation rate of bees can have a detrimental effect on fruit production, ultimately reducing crop yield [43]. The honey bee *A. mellifera* demonstrates a low tripping rate of flowers, but this is compensated by a high frequency of visits, resulting in greater pollination effectiveness. However, a saturation point may be reached where pollination effectiveness no longer improves despite an increase in visit frequency [44]. Contrary results have also been obtained from previous studies, which reported no effects on yield from supplementary treatments, regardless of whether pollinators were present or absent in the soybean crop [45,46]. One reason for the low contribution of supplementary pollination may be the insufficient number of honey bees visiting the crop. Another reason could be the low yield potential of the cultivars used for the experiment. However, installing honey bee colonies in watermelon fields can have a negative impact on native insect pollinators, which are essential for sustainable crop practices [18]. A high abundance of honey bees could reduce native pollinator populations. However, if there is already a low abundance of solitary bees causing a pollination deficit, supplementary pollination using *A. mellifera* is the only feasible option available to increase seed production in alfalfa [44].

A single visit by pollinator species is an essential criterion for assessing the efficacy of native insect pollinators [37]. In our study, *Megachile* sp. gave the highest number of seeds, and seed and pod weights in a single visit, followed by *A. mellifera*, the supplemented bee. A sufficient population of effective pollinators is necessary to pollinate crops properly [26,47]. Previous studies have reported the effectiveness of a single visit of *A. mellifera* [18]. Solitary bees are difficult to manage under agricultural intensification practices due to their complex seasonal behaviour and low availability of suitable nesting sites. However, low abundance of the most efficient pollinators can lead to a pollination deficit [48]. The reduction of pollination in crops can lead to decreased yield production [49,50]. To fulfil this pollination deficit in the crop, supplementary honey bee pollination is necessary to ensure that an adequate number of insect pollinators are available in the field [51].

In this study, bees were identified as the most abundant pollinators in the field, followed by syrphid flies, butterflies, and wasps. In the first year of the study, the abundance of *M. cephalotes* and *M. hera* was higher than that of *A. mellifera,* likely due to the presence of other crops, such as sponge gourd, cucumber, and other cucurbits, which attracted bees from our study fields. In the second year, we observed a significantly higher abundance of *A. mellifera*. This may be due to climatic factors, such as higher rainfall in the second year, which reduced flight activity and led to a low availability of other bees attracted to flowers. The presence of pollinators in sufficient density is essential to achieve adequate pollination, and this can be managed by supplementing with *A. mellifera* [44]. However, honeybees physically disturb and exclude smaller pollinator species from flowers [52]. In several earlier investigations, solitary bees were seen in more significant numbers at alfalfa flowers [39]. It has been noted that other crops, such as canola [37], pumpkin [53], sponge gourd [54], and radish, have larger abundances of *A. mellifera* and solitary bees [14].

In alfalfa, effective pollination is essential for seed production due to its unique floral morphology, which requires a mechanical action known as “tripping” to expose reproductive structures. Our results show a correlation between flower tripping and seed parameters, which proves the dependence of alfalfa on efficient pollinators [26]. However, the ability of a pollinator to trip alfalfa flowers is influenced by several morphological, behavioural, and ecological traits. Specialized bees like *Megachile* species are more efficient as compared to the honey bees, which are less efficient due to sudden strike of anthers on their head during the alfalfa flower tripping process [14]. Taking into consideration the tripping efficiency of different pollinator species, we found that *M. cephalotes* and *M. hera* showed the best performance, while *A. mellifera* demonstrated moderate tripping efficiency. Moreover, *A. florea* showed a nectar-feeding habit without tripping a flower. *A. florea* bees inserted their proboscis to feed on the nectar from the base of a flower, and the flower remained mostly untripped [55]. The low tripping rate of alfalfa by *A. florea* bees was due to this type of behaviour. These findings highlight the importance of selecting efficient pollinator species and optimizing their densities to maximize pollination success and seed yield in alfalfa cultivation.

## 5. Conclusions

Pollinator management, particularly through the supplementation of honey bee colonies, played a key role in increasing pollinator abundance and consequently improved seed weight and overall reproductive success of alfalfa plants. The lack of significant differences in seed weight between the highly supplemented and non-supplemented blocks draws attention to the nuances of pollination dynamics. This indicates that optimal conditions for pollination might extend beyond the number of pollinators present and could involve the interactions and behaviour of individual species—a critical consideration for future agricultural practices. The visitation rate analyses corroborated these findings, revealing that species like *Amegilla* sp. and *M. cephalotes* were significantly more active, thereby potentially influencing pollination outcomes positively. Furthermore, the tripping behaviour exhibited by different pollinator species provided a fascinating layer of complexity; *M. cephalotes* and *M. hera* demonstrated superior tripping efficacy, indicating their crucial role in ensuring adequate flower fertilisation. The integration of both managed and wild pollinators into agricultural systems could underpin sustainable practices that bolster biodiversity while optimising crop yields.

## Figures and Tables

**Table 1:** Correlation matrix showing Pearson’s correlation coefficients (r) and corresponding significance levels *p*-values for showing the relationships between flower tripping by pollinators and seed yield parameters (pod weight, number of seeds, and seed weight).

Variables	Flower Tripping	Pod Weight (g)	No. Seeds	Seed Weight (g)
**Flower tripping**				
**Pod weight (g)**	0.2575 (*p* = 0.0433)			
**No. Seeds**	0.3605 (*p* = 0.0040)	0.4812 (*p* < 0.001)		
**Seed weight (g)**	0.3347 (*p* = 0.0078)	0.5286 (*p* < 0.001)	0.8907 (*p* < 0.001)	

## Data Availability

Data will be available on request.
